# Is casting of displaced paediatric distal forearm fractures non-inferior to reduction under general anaesthesia? Study protocol for a pragmatic, randomized, controlled non-inferiority multicentre trial (the casting trial)

**DOI:** 10.1186/s13063-024-08253-z

**Published:** 2024-06-27

**Authors:** Katrine Rønn Abildgaard, Peter Buxbom, Ole Rahbek, Martin Gottliebsen, Per Hviid Gundtoft, Bjarke Viberg, Stig Brorson

**Affiliations:** 1 Department for Orthopaedic Surgery, Centre for Evidence-Based Orthopaedics, Zealand University Hospital, Køge, Denmark; 2 Department of Orthopaedics, Children’s Orthopaedics, Aalborg University Hospital, Aalborg, Denmark; 3 Danish Paediatric Orthopaedic Research, University Hospital Aarhus, Aarhus, Denmark; 4 Department of Orthopaedic Surgery and Traumatology, Odense University Hospital, Odense, Denmark

**Keywords:** Forearm injuries, Wrist injuries, Salter-Harris fractures, Closed fracture reduction, Child, Bone remodelling

## Abstract

**Background:**

Treatment of displaced distal forearm fractures in children has traditionally been closed reduction and pin fixation, although they might heal and remodel without surgery with no functional impairment. No randomized controlled trials have been published comparing the patient-reported functional outcome following non-surgical or surgical treatment of displaced paediatric distal forearm fractures.

**Methods:**

A multicentre non-inferiority randomized controlled trial. Children aged 4–10 years with a displaced distal forearm fracture will be offered inclusion, if the on-duty orthopaedic surgeon finds indication for surgical intervention. They will be allocated equally to non-surgical treatment (intervention) or surgical treatment of surgeon’s choice (comparator). Follow-up will be 4 weeks and 3, 6, and 12 months. The primary outcome is the between-group difference in 12 months QuickDASH score. We will need a sample of 40 patients to show a 15-point difference with 80% power.

**Discussion:**

The results of this trial may change our understanding of the healing potential of paediatric distal forearm fractures. If non-inferiority of non-surgical treatment is shown, the results may contribute to a reduction in future surgeries on children, who in turn can be treated without the risks and psychological burdens associated with surgery.

**Trial registration:**

www.clinicaltrials.gov (ID: NCT05736068). Date of registry: 17 February 2023.

**Supplementary Information:**

The online version contains supplementary material available at 10.1186/s13063-024-08253-z.

## Background and rationale

Paediatric distal forearm fractures (DFFs) are common and account for 25–30% of all fractures in children [1, 2]. In Denmark, the incidence among 4–10-year-old children is approximately 900/100,000 persons, corresponding to 3500 injuries per year [3], of which nearly half are treated surgically [4]. The most common treatment of displaced paediatric DFFs is currently closed reduction under general anaesthesia with or without pin fixation (or in rare cases plate and screw fixation), followed by immobilization in a cast [5]. However, surgery may have detrimental effects, such as fear, anxiety, and complications related to surgery. Eliminating these effects are desirable for the individual child and family. Children’s bones, and in particular the metaphysis and physis, have a unique ability to heal and remodel throughout the growth period [6], making a non-surgical approach a possible alternative. If more distal forearm fractures could be treated non-surgically in the future, the surgical capacity and allocated surgeons could be prioritized to patients of higher needs. As these fractures would be carried out in an outpatient setting, there will be no need for hospitalizations, benefitting both the individual patient and the health care system. In addition, from a socioeconomic perspective, re-prioritizing the use of healthcare resources could result in considerable financial savings [7–9].

Numerous studies, including small cohort studies, randomized controlled trials (RCTs), and case series, have found pin fixation advantageous in achieving anatomic reduction and avoiding re-displacement [10–16]. However, it is unknown whether patient-reported outcomes benefit from anatomic reduction and stabilization, as most studies use only radiographic or objective measures such as range of motion (ROM). During the last 20 years, only four studies have been published, investigating non-surgical treatment of displaced DFFs in prepubertal children. These include one Finish case–control study [17], one British prospective cohort study [18], and two retrospective case series from Finland and Hawaii, respectively [8, 19]. These studies do however agree that displaced DFFs might heal well without reduction and that most fractures will remodel almost to the anatomical position with no functional impairment within a year or two.

There are currently two other ongoing RCTs comparing non-surgical treatment to surgical treatment. One is a large study in England, the CRAFFT study [20], randomizing 750 children nationwide with displaced distal forearm fractures to non-surgical or surgical treatment comparable to the interventions in this trial. The other RCT is conducted in Finland [21], randomizing 60 children with overriding distal forearm fractures to surgical treatment or non-surgical treatment, where finger trap traction is used to align only angulation but not dorsal displacement or shortening. To the best of our knowledge, no RCTs have been published yet, and none of the existing studies report outcomes from the patient’s perspective.

### Objective

The objective of this trial is to compare patient-reported outcome measures (PROMs) assessing function, quality of life, and pain following non-surgical versus surgical treatment of displaced DFFs in 4–10-year-old children.

### Trial design

A pragmatic, randomized, controlled non-inferiority multicentre trial with two parallel groups allocated 1:1 by block randomization. Primary outcome is patient-reported function after 1 year.

## Methods

This trial protocol was written according to the SPIRIT guidelines (Additional file 1) [22].

### Study setting

The trial will be conducted at orthopaedic surgery departments at four Danish university hospitals in Køge, Aarhus, Aalborg, and Odense.

### Eligibility criteria

**Fig. 1:**
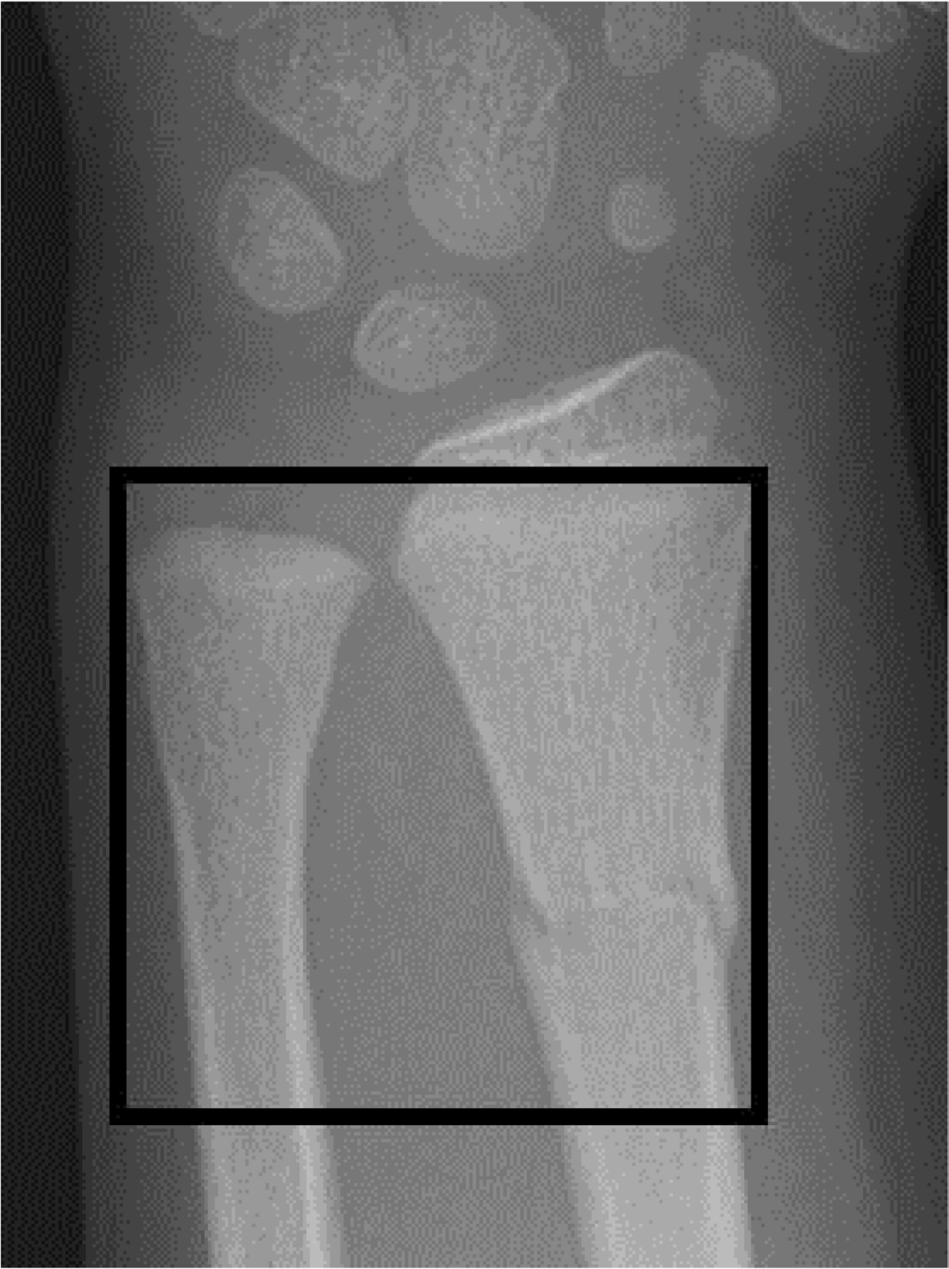
The metaphysis defined by the AO square with its sides being the same lengths as the width of radius and ulna at the level of the radius growth plate

For definition of the metaphysis, we will use the AO classification for children, as illustrated in Fig. 1 [23].

### Inclusion criteria

- Children 4–10 years of age with open physes
- Displaced fractures in the distal metaphyseal radius with or without concomitant ulna fracture (S52.5 (distal radius) and S52.6 (distal radius and ulna)), including extra-articular physeal fractures (Salter-Harris (SH) I–II)
  - ◦ Overriding fractures or
  - ◦ Angulated fractures of 20–40°
- The on-duty surgeon finds reduction under anaesthesia with or without fixation indicated

### Exclusion criteria

- Open fractures
- Nerve or vascular affection
- Intraarticular fractures including SH III–V
- Ulnar physeal fractures
- Polytrauma
- Concomitant ipsilateral or contralateral upper extremity fractures (except distal ulna fracture)
- Pathologic fractures
- The injury is > 7 days old
- Other conditions that may affect bone healing

### Informed consent

A child presenting with a displaced DFF is screened for eligibility. If the eligibility criteria are fulfilled, the surgeon will briefly introduce the project and ask the parents/guardians to meet in the outpatient clinic the following day. Here, the local investigator will provide both oral and written information including trial participant’s rights and a consent form (Additional file 6). The information will be adapted to the child’s ability to understand the project and its importance for them. After a reflection period, the parents/guardians will be asked for written consent.

## Interventions

A cast will be applied in the emergency room as standard procedure. Children will be randomly allocated 1:1 to:

1. Non-surgical treatment (intervention): No reduction. Cast optimization if necessary
2. Surgical treatment (comparator): Reduction under general anaesthesia with or without pin fixation of surgeons’ choice followed by cast immobilization

The cast will be removed after 4 weeks if radiological signs of healing (callus formation or bone bridges). The casting will be prolonged another 2 weeks if uncertainty of healing. If the initial cast was an above elbow cast, it will be changed to a below elbow cast at this point.

### Discontinuation

Compartment syndrome and carpal tunnel syndrome has been described in relation to distal forearm fractures. However, the evidence-base is very limited and is primarily based on case reports in fractures after reduction [24, 25]. Thus, as these complications appear after surgery, we expect a lower incidence in the non-surgical group.

### Adherence to intervention

It is not possible to cross-over from surgical to non-surgical treatment. Although the opposite is possible, efforts will be made to avoid cross-overs, and changing from non-surgical to surgical treatment will be considered a drop-out. Patients dropping out will be scheduled for usual care, i.e. surgery as long as the fracture has not healed, and preferably within 5 days from injury for physeal fractures and within 10 days for metaphyseal fractures.

### Protocol violation

- Loss to follow-up
- Health conditions precluding participation

The trial will be discontinued if unexpected and serious complications arise, and all participants will be notified and offered standard treatment.

### Insurance

Insurance of the trial is covered by The Danish Patients Compensation Fund.

## Outcomes

Patients will be followed up by 4 weeks and 3, 6, and 12 months. The surgeon may schedule the patient for an additional post-operative 1 week control if preferred. If needed, we will offer long-term follow-up of patients beyond the scope of this trial.

### Primary outcome measure

The primary outcome will be the PROM, Quick Disabilities of Arm, Shoulder and Hand (QuickDASH) [26], at 12 months. This 11-item questionnaire is a shortened version of the 30-item Disabilities of the Arm, Shoulder and Hand (DASH) [27]. The DASH was designed to help report the disability experienced by adults with upper-limb disorders covering different activities of daily living, and also monitoring changes over time.

The QuickDASH has, like the DASH, two four-item optional modules, work and sports/performing artists, which are scored separately. The QuickDASH and the DASH have comparable psychometric properties [28].

The patient rates each item (with help by parents if the patient is too young to self-report) according to the perceived degree of severity using a 5-point Likert scale. The overall score is transformed to a score between 0 and 100 (0 = no disability, 100 = maximum disability).

Although the QuickDASH was developed on adults, it has been used in several studies on children with different upper extremity fractures. Unfortunately, these studies report only total QuickDASH scores (mean (standard deviation (SD))) and *p*-values but do not take into account the clinical relevance of the differences [29–33].

We have identified four studies using anchor-based approach to determine the minimal clinically important difference (MCID) in adults [34–37]. Mintken et al. [37] reported an MCID of 8 on 101 adults with shoulder pain. Sorensen et al. [35] reported an MCID of 14 on 48 adults with non-operatively treated atraumatic hand and forearm conditions. Franchignoni et al. [34] reported an MCID of 15.9 on 266 adults with upper-limb musculoskeletal disorders, referred to inpatient and outpatient physiotherapy. In a similar population, Polson et al. [36] reported an MCID of 19 on 35 adults with musculoskeletal conditions to the upper extremity, referred to physiotherapy. We derive an MCID of 15 from the reported MCIDs, and since no MCID is reported in children’s cohorts, we assume it to be roughly comparable to adults.

To estimate an SD for the QuickDASH, we identified SDs in studies on children with upper extremity fractures. Quatman-Yates et al. [29] reported an SD of 19.3 in 149 children aged 8–12 years referred for outpatient rehabilitation following upper extremity injury. Ernat et al. [33] assessed 752 supracondylar fractures in 2–13-year-old children and the relationship between fracture classification and QuickDASH after 3 months (range 1–13 months). They reported SDs of 11.6 in Gartland II and 16.4 in Gartland III. Eguia et al. [32] did a cross-sectional survey 4.4 years (range 2–10 years) post-operatively on 508 children aged 3–8 years with a supracondylar humerus fracture treated with crossed or lateral pinning. They reported SDs of 5.8 and 8.8, respectively. Roper et al. [31] did a cross-sectional study 5 years post-injury on 30 children < 18 years old with closed or open Monteggia fractures and reported SDs of 6.1 and 8.8, respectively. Overall, there seems to be a tendency for the SD to decrease over time. Assuming these populations are roughly comparable to those of our study, the SD should be somewhere between 11.6 and 16.4 by 3 months (with a range up to approximately 1 year) and 5.8 and 8.8 by 4.4 years. From these assumptions, we conservatively estimate the SD to be 15, since our primary outcome at 12 months is closer to 3 months than 4.4 years.

### Secondary outcome measures

Secondary outcomes will include the following:

- QuickDASH (3 and 6 months)
- Health-related quality of life (HRQoL) using EQ-5D-Y [38, 39] (3, 6, and 12 months), and
- Pain using Wong-Baker Faces Pain Scale (WBS) [40] (3, 6, and 12 months)

#### EQ-5D-Y

The EQ-5D-Y is a child-friendly version of the EuroQol EQ-5D generic measure of HRQoL. It consists of two parts: the first part (the descriptive system) assesses health in five dimensions (mobility; looking after myself; doing usual activities; having pain or discomfort; feeling worried, sad or unhappy), each of which has three levels of response. This part of the EQ-5D-Y questionnaire provides a description of the respondent’s health by generating a health state profile. The second part of the questionnaire, the EQ VAS, consists of a visual analogue scale (VAS) on which the respondent rates their perceived health from 0 (the worst imaginable health) to 100 (the best imaginable health). We define an MCID of 10 EQ VAS points and SD = 20, based on a study including 3–6-year-old children, either healthy or with acute or chronic illness [41].

##### Wong-Baker Faces Pain Rating Scale

The WBS is a widely used self-reported tool to assess pain using a series of six facial expressions to illustrate the degree of pain [40]. A numerical value is assigned to each face, from 0 (no hurt) to 10 (hurts worst); thus, each face equates 2 points. It has been validated among children above the age of 3 with sickle cell anaemia and human immunodeficiency virus (HIV) infection as well as children undergoing venepuncture and minor surgery [42]. The WBS has an MCID of one face (2 points) [43].

### Explorative outcomes

#### Radiographs

The remodelling process will be evaluated by the axial alignment of radius on antero-posterior (AP) and lateral radiographs taken at 6 and 12 months. Angular malalignment in metaphyseal and physeal fractures is determined as the angle formed by the intersection of two lines parallel to the axis of the radius proximal and distal to the fracture site or growth plate. Physeal arrest will be recognized by the presence of focal bone density bridging across the normally lucent physis.

### Photographs

We will take photographs (AP and lateral views) at the time of injury, 4 weeks, and 3-, 6-, and 12-month visits to observe the cosmetic progress.

No systematic measurements will be made on radiographs or photographs, but they may support our primary and secondary outcomes and used for didactic purposes.

### Participant timeline

We will follow the Standard Protocol Items Recommendations for Interventional Trials (SPIRIT) (Table 1).

**Table 1:**
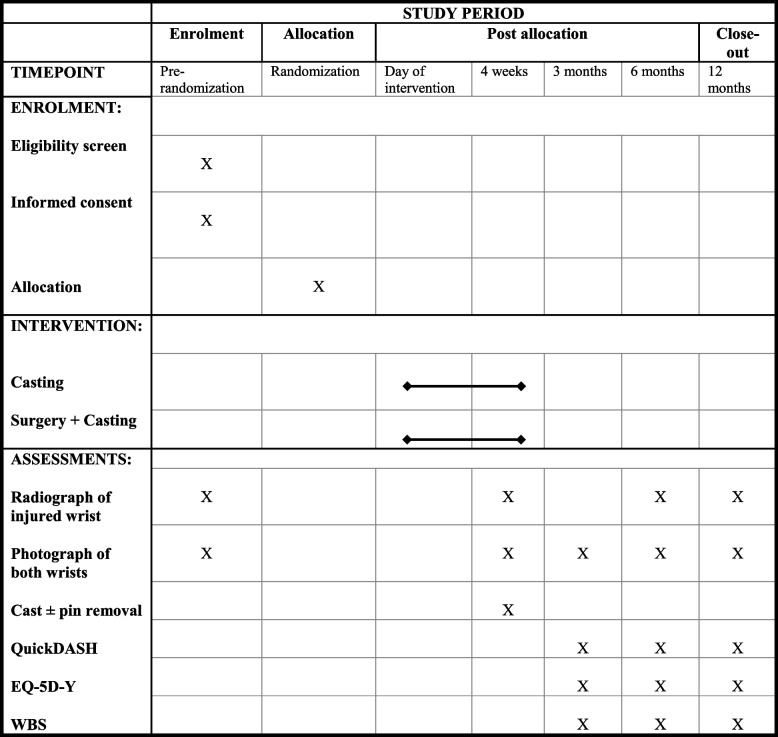
Standard Protocol Items Recommendations for Interventional Trials (SPIRIT) diagram. QuickDASH, Quick Disabilities of the Arm, Shoulder and Hand. EQ-5D-Y, EuroQol Youth version to measure Health-Related Quality of Life (HRQoL). WBS, Wong-Baker Faces Pain Rating Scale

### Sample size

We defined a non-inferiority margin as NIM = MCID = 15 QuickDASH points. Thereby, we will only reject the null-hypothesis, if the between-group difference is *both* more than NIM *and* clinically relevant. With an SD = 15 and type I error rate of 2.5%, 16 patients per group (32 in total) would be required for 80% power that the lower limit of a 95% two-sided confidence interval (CI) would be above the non-inferiority limit [44]. Allowing for 20% dropouts, the total sample size required is 40 patients (20 in each group).

### Recruitment

We will have four recruiting centres, thereby improving the rate of enrolment.

## Assignment of allocation

### Sequence generation

A person not otherwise involved in the study will generate a random allocation sequence in the R software with block randomization (70 blocks with shifting block sizes of 2, 4, and 6 in each block). The same person with no other user rights will upload the table in the software Research Electronic Data Capture (REDCap), and the table will be blinded to all other investigators.

### Concealment of allocation

The local investigator, with randomization rights assigned by the principal investigator (PI), will log into REDCap and irreversibly allocate patients into one of the two treatment groups. The local investigator is responsible for providing the allocated treatment by either scheduling the patient for surgery or applying a cast (or leaving the already applied cast).

## Blinding

It is not possible to blind the surgeon, the patient, or the parents/guardians to the treatment allocation. PROM scores and 6- and 12-month radiographs will be evaluated by persons blinded to treatment allocation and not otherwise involved in the study. Data analysis will be performed by an external biostatistician blinded to treatment allocation.

### Patient and public involvement

Three parents read and commented on the written information sheet, thereby reassuring that it was understandable and sufficiently informing.

## Data collection and management

### Data collection

We will collect baseline data and outcome measures by 4 weeks and 3, 6, and 12 months post-injury in REDCap (or paper forms if no REDCap access) (Additional file 2). Link to questionnaires will be sent by email 2 days prior to the appointment.

### Participant retention

Efforts will be made to inform patients of the importance of completing the entire study period. By receiving questionnaires electronically 2 days in advance, patients will be reminded of their appointment.

Patients may withdraw from the study for any reason at any time. Patients will be excluded from the trial if they fail to show up despite two phone call reminders. We will include data obtained until withdrawal in the statistical analysis.

### Data management

Approval from the Data Protection Agency of Region Zealand have been obtained (REG-099–2022) before trial commencement. Compliance with the General Data Protection Regulation (GDPR) and the Danish Data Protection Act will be ensured at all times. A data processing agreement has been signed with all recruiting centres. All completed paper forms will be stored in locked file cabinets with limited access before and after they are entered in REDCap (with two-factor authentication login). The REDCap setup ensures that randomization is only possible, if all baseline information is entered correctly. Other electronic participant information, if any, will be stored on a secured study-specific drive owned/managed by the PI. All data will be fully anonymized before publication.

## Statistical methods

Data analysis will be conducted by an external biostatistician. Descriptive statistics will be used to report demographic data.

Continuous variables will be reported by mean with SDs or median with interquartile range (IQR), depending on the distribution, and compared using *t*-test for normal distributed variables and Mann–Whitney *U* test for non-normal distributed variables. Categorical variables will be reported by numbers and percentage and will be compared using Pearson’s chi-square test.

Significance is set as *p*-value < 0.05.

### Primary outcome analysis

The primary endpoints will be reported as mean QuickDASH scores on a continuous scale between 0 and 100 and will be analysed by the per-protocol (PP) population and repeated, for sensitivity reasons, for the intention-to-treat (ITT) population. Non-inferiority will be concluded only if both PP and ITT analyses show non-inferiority.

The between-group difference in mean QuickDASH score by 12 months will be compared using linear mixed effects models (LMM).

Non-inferiority will be concluded if the lower end of the 95% CI is above the NIM of 15 QuickDASH points.

### Secondary outcome analysis

The between-group difference in mean QuickDASH score by 3 and 6 months will be compared as above for the PP population. The between-group difference in mean EQ VAS scores on a continuous scale between 0 and 100 will be analysed by 3, 6, and 12 months and compared using LMM. The between-group difference in WBS scores between 0 and 10 points (from 6 answer options corresponding 0, 2, 4, 6, 8, and 10 points) will also be analysed by 3, 6, and 12 months and compared using LMM.

A health profile will be generated for each patient using the EQ-5D-Y descriptive system. Summary statistics will be derived, including numbers and proportions of patients reporting each level of severity in each EQ-5D-Y dimension in each visit.

Observed and patient-reported adverse events will be reported with descriptive statistics, grouping patients according to received treatment.

### Interim analyses

We plan to perform an interim analysis when 20 patients (10 in each arm) have had their 6 months follow-up. The results from the interim analysis will not be published separately but will be part of the final report.

### Handling of missing data

LMM will be used to deal with missing values, assuming the dropout mechanism is missing at random (MAR). We distinguish between item-wise missing (more than one, but not all, answers in a questionnaire are missing) and case-wise missing (all answers in a questionnaire are missing). Case-wise missing will be addressed using LMM. Incomplete questionnaires with item-wise missing will be addressed by multiple imputation, if the number of questionnaires excluded due to missing items exceeds 5%.

## Monitoring

### Trial steering committee

The authors of this protocol article comprise the trial steering committee. They are responsible for conduction of the trial at each site. Smaller day-to-day information or challenges are handled via email correspondence, mainly between the PI and local investigators. Status of the trial or other important topics are discussed at 2–3 physical or virtual meetings per year.

### Data monitoring

A data monitoring committee (DMC) is unnecessary as this is an open label trial with continuous access to all data, and the steering committee will continuously evaluate the outcomes.

### Harms

The local investigators will monitor, and report to the PI, all observed and patient-reported adverse and unexpected events, including infections (superficial, deep), iatrogenic nerve or vascular damage, neuropraxia, uncomfortable scar tissue formation, and any cast problem (including change of cast) as well as events not mentioned but otherwise considered important. For the surgical group, we will also report re-displacements leading to re-operations and all-cause re-operations. The PI will evaluate all events to classify their severity and relatedness to the treatment. All serious adverse events (The World Health Organization (WHO) definition) related to the treatment will be reported to the Research Ethics Committee for Region Zealand within 7 days from the time of the event.

Radiation exposure does not exceed that of patients outside the study. Participation may even result in less radiation exposure as all included patients, initially intended for surgical treatment with the use of perioperative fluoroscopy, will instead be treated non-surgically, thereby minimizing the amount of radiation.

### Protocol amendments

Update on any protocol changes during the study period will be given by the PI to all recruiting centres as well as trial participants for whom it may be relevant. Substantial modifications such as changed eligibility criteria, follow-up visits, outcomes, or statistical analysis will be registered at www.clinicaltrials.gov, and a supplementary protocol will be submitted to the Research Ethics Committee in Region Zealand.

## Dissemination

The trial protocol is preregistered at www.clinicaltrials.gov. All results from the study—both positive, negative, and inconclusive—will be published in a relevant, international, scientific peer-reviewed journal. The PI will ensure publication with authorship following the guidelines of the International Committee of Medical Journal Editors (ICMJE) as well as the Consolidated Standards of Reporting Trials (CONSORT) guidelines for the reporting of parallel group randomized trials. Results will be presented at relevant national and international conferences, e.g. the Danish Orthopaedic Association. A website, www.thecastingtrial.com, is linked to the study; all relevant material and results will be available here.

### Ethical considerations

Today, most children with displaced distal forearm fractures are treated surgically under general anaesthesia with closed reduction and pin fixation. Besides the fact that surgery and anaesthesia can be stressful for the child and family, there are risks associated with surgery. These include infection, damage to the surrounding vessels, tendons and nerves, and scar tissue formation. Re-displacements are common (up to 50%), and re-operations with re-reduction with or without pin fixation may be necessary in up to half of these cases [11, 15]. Following pin fixation, a subsequent procedure (though most often without anaesthesia) is needed to remove the pins again. The child undergoing surgery will be exposed to a relatively large amount of radiation (almost threefold higher than children being treated non-surgically) due to the use of fluoroscopy in the operating room (OR) [45, 46].

In less than 0.6% of cases, a later corrective surgery will be needed if the bone remains misaligned, regardless of surgical or non-surgical treatment [47]. However, this is particularly rare in the population included in this study, as children of this age still have a great remodelling potential.

Each child in this RCT will have a 50% chance of avoiding surgery and the associated inconveniences and potential complications. In return, some children may experience some forearm deformity which we expect is mainly a cosmetic issue and has minimal or no impact on the function. If the results of this study demonstrate non-inferiority of non-surgical treatment compared to surgical treatment, it opens up the possibility of treating up to 1800 children per year non-surgically. In summary, we believe that the disadvantages associated with perhaps having a temporarily misaligned forearm are outweighed by the benefits of avoiding surgery.

## Discussion

We aimed to assess the patient-reported functional outcome after 1 year using a PROM. Unfortunately, no PROM has been developed and validated for children with upper extremity injuries. The QuickDASH has been found a reliable and valid instrument among 8–18-year-old children with upper extremity injuries [29]. It has several advantages: (1) it is short, thus reducing time consumption; (2) a Danish translation is already available; and (3) the questionnaire is feasible for children to complete despite 1–2 challenging questions.

Regarding children, there is no definition of an MCID of the QuickDASH. We defined ourselves an MCID based on a series of adult studies, assuming it to be the same in children. Future goals should be to develop a PROM on this population and, through anchor-based approaches, define a more precise MCID.

Although our study is somewhat comparable to the CRAFFT [20] study and the Finish study by Laaksonen et al. [21], the three studies also differ in either population, intervention, and/or outcome measures. The CRAFFT study includes a similar, yet larger (*n* = 750), population regarding age and fracture types. They use stratified randomization with stratification factors including centre, fracture severity and location, and patient age. We did not use stratification for several reasons: (1) we expected girls and boys in this age to have similar remodelling capacity, (2) the study is not powered to ensure equal distribution of covariates, and (3) the steering committee represents 4 out of 5 regions in Denmark, and treatment management is almost identical across regions using the same clinical guidelines. Thus, we expect no or minimal clustering effect due to treatment differences across centres. The intervention in CRAFFT is, as in our study, non-surgical treatment compared to surgical. However, they allow also reduction under conscious sedation, which we do not in our protocol. The primary outcome is patient-reported function measured by Patient-Reported Outcomes Measurement Information System Upper Extremity (PROMIS UE) by 3 months and a wide range of secondary outcomes measured by 6 weeks to 3 years follow-up including WBS, EQ-5D-Y, VAS cosmesis, and healthcare resource use. Our primary outcome is by 12 months as this is when we expect the fractures to be remodelled enough for the forearm to have regained its normal function. In relation to CRAFFT, they have conducted a qualitative study asking patients and their parents about their experience of having the injury and their thoughts about participating in a clinical trial. We used some of the information from this qualitative study to form our protocol. For instance, we decided to give information about the study the following day instead of in the emergency room (ER) as parents explained they were not able to receive the information because the situation in the ER were too stressful. Laaksonen et al. [21] will include all 0–10-year-old children (*n* = 60), but only with overriding fractures. Their intervention is to cast the fracture after finger trap traction in order to regain forearm axis, but not dorsal displacement and shortening. Their primary outcome is active forearm rotation, flexion, and extension in injured versus uninjured forearm. Secondary outcomes include radiographs, QuickDASH, Paediatric Quality of Life Inventory (PedsQL), and overall satisfaction. Although the study by Laaksonen et al. differ most from our study and CRAFFT, we have in common to investigate the potential of fracture remodelling in younger children in order to reduce the surgical burden.

In recent years, there have been a number of initiatives regarding treatment of other medical conditions that require health care providers to adapt to new and, in some cases, considerably altered treatments. In particular, researchers have focused on health education of both health providers and patients in order to improve treatment and quality of life [48]. In relation to our study, non-surgical treatment of displaced distal forearm fractures may seem counter-intuitive to surgeons as well as patients and parents. Patient/parent education and thorough information about the benefits and risks of both surgical and non-surgical treatment are therefore essential to ensure the best conditions for shared decision making.

## Trial status

Protocol version 3, June 12, 2023. Recruitment began: August 28, 2023. Expected recruitment completion: May 2025.

### Supplementary Information

Additional file 1. SPIRIT checklist.

Additional file 2. REDCap instruments (questionnaires and forms for baseline information and control visit information).

Additional file 3. Original and English translations of funding documentation.

Additional file 4. Original and English translation of guarantee for covering of salary.

Additional file 5. Original and English translation of ethical approval.

Additional file 6. Consent form.

## Data Availability

The PI is the owner of the full data set. Each site has access to its own data only.

## Abbreviations

AO: Arbeitsgemeinschaft für Osteosynthesefragen
AP: Antero-posterior
CI: Confidence interval
CONSORT: Consolidated Standards of Reporting Trials
CRAFFT: Children’s Radius Acute Fracture Fixation Trial
DASH: Disabilities of Arm, Shoulder and Hand
DFF: Distal forearm fracture
DMC: Data monitoring committee
GDPR: General Data Protection Regulation
HIV: Human immunodeficiency virus
HRQoL: Health-related quality of life
ICMJE: International Committee of Medical Journal Editors
IQR: Interquartile range
ITT: Intention-to-treat
LMM: Linear mixed effects model
MAR: Missing at random
MCID: Minimal clinically important difference
MeSH: Medical Subject Headings
NIM: Non-inferiority margin
OR: Operating room
PedsQL: Paediatric Quality of Life inventory
PI: Principal investigator
PP: Per protocol
PROM: Patient-reported outcome measure
PROMIS UE: Patient-Reported Outcomes Measurement Information System Upper Extremity
QuickDASH: Quick Disabilities of Arm, Shoulder and Hand
RCT: Randomized controlled trial
REDCap: Research Electronic Data Capture
ROM: Range of motion
SD: Standard deviation
SH: Salter-Harris
SPIRIT: The Standard Protocol Items: Recommendations for Interventional Trials
VAS: Visual analogue scale
WBS: Wong-Baker Faces Pain Rating Scale
WHO: The World Health Organization
