# Supplementary material for: Is casting of displaced paediatric distal forearm fractures non-inferior to reduction under general anaesthesia? Study protocol for a pragmatic, randomized, controlled non-inferiority multicentre trial (the casting trial)
Source: Trials. 2024 Jun 27;25:420. doi: 10.1186/s13063-024-08253-z (PMC11212181; doi:10.1186/s13063-024-08253-z)
Supplement: Supplementary file 1 — Additional file 1. SPIRIT checklist. [file 13063_2024_8253_MOESM1_ESM.pdf]

Dataregistrering før randomisering

Study ID ______________________

Deltagerinformation

Fornavn ______________________

Mellemnavn ______________________

Efternavn ______________________

Fødselsdato ______________________
(DD-MM-YYYY)

CPR-nr ______________________
(Uden bindestreg)

E-mail adresse ______________________

Køn
- ◯ Pige
- ◯ Dreng

Alder på inklusionstidspunktet ______________________

Inklusionskriterier

Har patienten åben epifyseskive på røntgen?
- ◯ Nej
- ◯ Ja

AO-klassifikation benyttes til vurdering af, hvorvidt frakturen er metafysær eller diafysær.

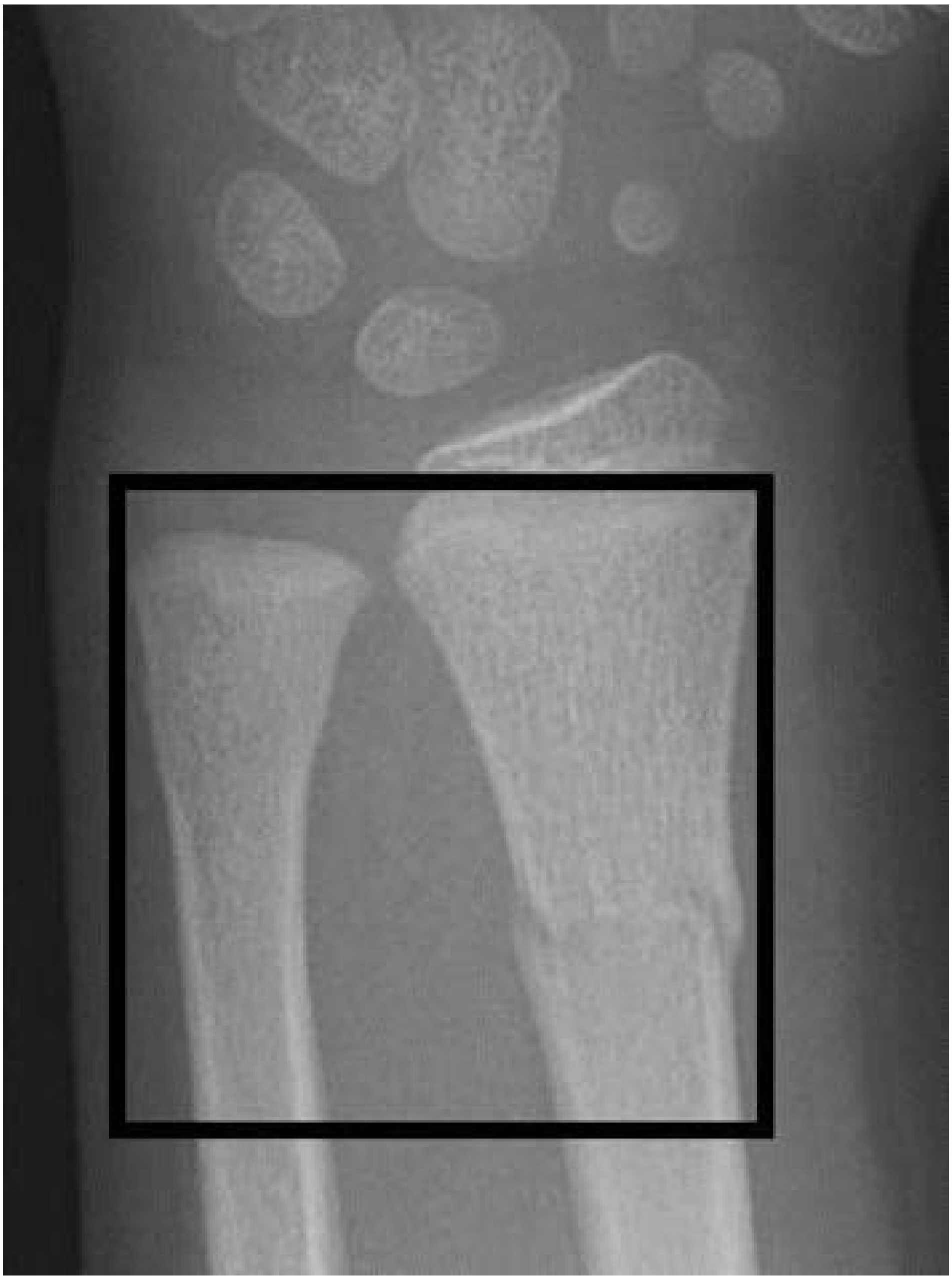

Ud fra denne klassifikation, er frakturen da metafysær?	◯ Nej<br>◯ Ja
Hvis frakturen ikke er metafysær, er den da en epifysiolyse?	◯ Nej<br>◯ Ja
Salter Harris klassifikation:	◯ 1<br>◯ 2<br>◯ 3<br>◯ 4<br>◯ 5
Er frakturen overridende?	◯ Nej<br>◯ Ja
Vinkler frakturen mellem 20-40 grader?	◯ Nej<br>◯ Ja

Angiv målt vinkel:	______________________ (1 decimal)
Har vagthavende kirurg fundet indikation for reposition i GA?	◯ Nej<br>◯ Ja

Eksklusionskriterier

Er det en åben fraktur?	◯ Nej<br>◯ Ja
Er der kar- eller nerveskade (gælder ikke paræstesier)?	◯ Nej<br>◯ Ja, karskade<br>◯ Ja, nerveskade
Er patientens fraktur del af et multitraume?	◯ Nej<br>◯ Ja
Har patienten samtidig anden fraktur i samme eller modsatte arm (undtaget distal ulna fraktur)?	◯ Nej<br>◯ Ja
Mistanke om patologisk fraktur?	◯ Nej<br>◯ ja
Er frakturen mere end 7 dage gammel?	◯ Nej<br>◯ Ja
Lider patienten af andre sygdomme?	◯ Nej<br>◯ Ja
Hvilke(n)?	______________________
Skønnes patientens sygdom at kunne påvirke knogleheling?	◯ Nej<br>◯ Ja
Tager patienten medicin?	◯ Nej<br>◯ Ja
Hvilke(n)?	______________________ (Type, dosis, frekvens)
Skønnes medicinen at kunne påvirke knogleheling?	◯ Nej<br>◯ Ja

Samtykke

Er der indhentet samtykke?	◯ Nej<br>◯ Ja
Dato samtykkeerklæring er underskrevet	____________________<br>(DD-MM-YYYY)

Foto af underarme

Billeder af børnenes underarme tages tilnærmelsesvist forfra og fra siden, som vist på billederne her:

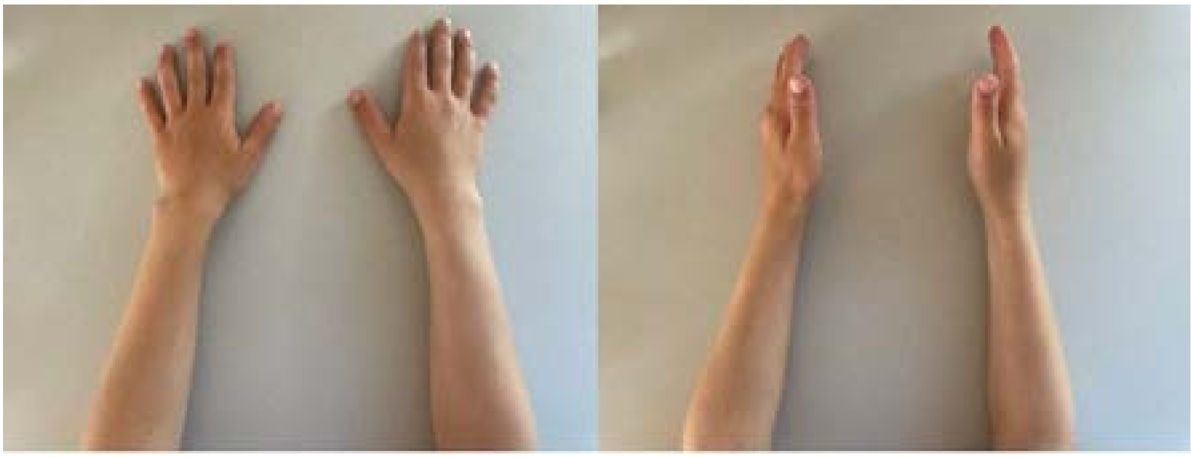

1) Upload venligst billeder af patientens underarme
(billede taget forfra/ AP)

2) Upload venligst billeder af patientens underarme
(billede taget fra siden/lateral)

Ekstra besøg før afbandagering

1) Dato for besøg

____

2) Årsag til ekstra besøg:

____
(Beskriv kortfattet årsag til ekstra besøg, f.eks. manglende heling, infektion, sårheling etc.)

3) Her kan uploades billede af sår, infektion e.lign. hvis det er relevant for dette besøg.

4) Her kan uploades billede af sår, infektion e.lign. hvis det er relevant for dette besøg.

5) Her kan uploades billede af sår, infektion e.lign. hvis det er relevant for dette besøg.

Kontrol i forbindelse med afbandagering

Dato for afbandagering: ______________________

Er der observeret/rapporteret komplikationer?
- ◯ Nej
- ◯ Ja

Sæt kryds i én eller flere
- ☐ Neuropraxi
- ☐ Gipsegener
- ☐ Andet

Hvilke(n)? ______________________

Sæt kryds i én eller flere
- ☐ Overfladisk infektion
- ☐ Dyb infektion
- ☐ Iatrogen nerveskade
- ☐ Iatrogen karskade
- ☐ Neuropraxi
- ☐ Ubehagelig arvæv
- ☐ Gipsegener
- ☐ Frakturskred
- ☐ Andet

Hvilke(n)? ______________________

Her kan uploades billede af sår, infektion e.lign. hvis det er relevant for dette besøg.

Her kan uploades billede af sår, infektion e.lign. hvis det er relevant for dette besøg.

Her kan uploades billede af sår, infektion e.lign. hvis det er relevant for dette besøg.

Har der været behov for re-operation?
- ◯ Nej
- ◯ Ja

Af hvilken årsag? ______________________

Dato for planlagt/udført operation ______________________

Spørgeskema om funktion i forbindelse med håndledsbrud

Kære [baseline_arm_1][fornavn].

I dette spørgeskema stiller vi dig spørgsmål om dine symptomer og din evne til at udføre visse aktiviteter.

Vær venlig at svare på hvert eneste spørgsmål ved at markere det svar, der passer bedst til din tilstand i den forløbne uge.

Hvis du ikke har haft lejlighed til at udføre en bestemt aktivitet i den forløbne uge, beder vi dig angive det svar, du mener ville dække bedst.

Det er uden betydning, hvilken hånd eller arm du anvender til at udføre aktiviteten; dit svar skal afspejle din evne til at udføre selve handlingen, uanset hvordan du gør det.

Bemærk! Når du kommer til det valgfrie arbejdsmodul, skal du betragte spørgsmålet "Arbejder du?" som "Går du i skole?", og "Angiv venligst hvad dit arbejde består i:" som "Hvilket klassetrin?". De følgende spørgsmål besvares således ud fra, hvordan du klarer at gå i skole.

Vurder venligst, hvordan din evne til at udføre følgende handlinger har været i den forløbne uge ved at markere det svar, der passer bedst.

	Ikke vanskeligt	Lidt vanskeligt	Noget vanskeligt	Meget vanskeligt	Umuligt
1. Åbne et (marmelade)glas med stramt låg.	○	○	○	○	○
2. Udføre tungt husarbejde (fx vaske vægge, vaske gulve).	○	○	○	○	○
3. Bære en indkøbspose eller en mappe.	○	○	○	○	○
4. Vaske dig selv på ryggen.	○	○	○	○	○
5. Bruge en kniv til at skære mad ud.	○	○	○	○	○
6. Fritidsaktiviteter, som sender en vis kraft eller stød gennem din arm, skulder eller hånd (fx golf, slag med hammer, tennis, osv.).	○	○	○	○	○
7. Hvor vanskeligt har det været for dig i den forløbne uge, at omgås familie, venner, naboer og grupper pga. din arm, skulder eller hånd?	○	○	○	○	○
8. Har du i den forløbne uge været hæmmet i at udføre dit arbejde eller andre gøremål pga. din arm, skulder eller hånd?	○	○	○	○	○

Vær venlig at angive sværhedsgraden af følgende symptomer i den forløbne uge.

	Ingen	Lidt	En del	Svær	Ekstrem
9. Smerte i din arm, skulder eller hånd når du laver noget bestemt.	○	○	○	○	○
10. Prikken i din arm, skulder eller hånd.	○	○	○	○	○

	Ikke vanskeligt	Lidt vanskeligt	Noget vanskeligt	Meget vanskeligt	Så vanskeligt at det forhindrer mig i at sove
11. Hvor vanskeligt har det i den forløbne uge været for dig, at sove pga. smerter i din arm, skulder eller hånd? (sæt cirkel om tallet)	○	○	○	○	○

ARBEJDSMODUL (VALGFRIT)

De følgende spørgsmål drejer sig om påvirkningen af din arbejdsevne pga. din arm, skulder eller hånd (inklusive husarbejde, hvis det er din hovedbeskæftigelse).

Arbejder du?
- ○ Nej, jeg arbejder ikke
- ○ Ja, jeg arbejder

Angiv venligst hvad dit arbejde består i: ______________________________

Markér venligst hvad der bedst beskriver din fysiske formåen i den forløbne uge. Havde du vanskeligt ved at:

	Ikke vanskeligt	Lidt vanskeligt	Noget vanskeligt	Meget vanskeligt	Umuligt
1. Bruge din sædvanlige fremgangsmåde i dit arbejde?	○	○	○	○	○
2. Udføre dit sædvanlige arbejde pga. smerter i din arm, skulder eller hånd?	○	○	○	○	○
3. Udføre dit arbejde så godt, som du gerne ville?	○	○	○	○	○
4. Udføre dit arbejde på den tid du plejer?	○	○	○	○	○

MODUL FOR SPORTSFOLK OG UDØVENDE KUNSTNERE (VALGFRIT)

De følgende spørgsmål drejer sig om, hvor stor en betydning dit arm-, skulder eller håndproblem har, når du spiller dit instrument, udøver din idræt eller begge dele. Hvis du dyrker mere end en sporstgren eller spiller mere end et instrument (eller begge dele), så svar venligst på grundlag af den aktivitet, som er vigtigst for dig.

Dyrker du sport eller spiller på et instrument?

- ◯ Nej, jeg dyrker ikke sport eller spiller på et instrument
- ◯ Ja, jeg dyrker sport eller spiller på et instrument

Angiv venligst den sportsgren eller det instrument, som er vigtigst for dig: ______________________

Markér venligst hvad der bedst beskriver din fysiske formåen i den forløbne uge. Havde du vanskeligt ved at:

	Ikke vanskeligt	Lidt vanskeligt	Noget vanskeligt	Meget vanskeligt	Umuligt
1. Bruge din sædvanlige fremgangsmåde når du spiller dit instrument eller dyrker din idræt?	◯	◯	◯	◯	◯
2. Spille dit instrument eller dyrke din idræt pga. smerter i din arm, skulder eller hånd?	◯	◯	◯	◯	◯
3. Spille dit instrument eller dyrke din idræt så godt som du gerne ville?	◯	◯	◯	◯	◯
4. Bruge den tid du plejer på at øve dig eller spille dit instrument / træne eller dyrke din idræt?	◯	◯	◯	◯	◯

Spørgeskema om livskvalitet i forbindelse med håndledsbrud

Kære [baseline_arm_1][fornavn].

I dette spørgeskema vil vi undersøge din livskvalitet relateret til dit helbred.

Vær venlig at svare på hvert eneste spørgsmål ved at markere det udsagn, der passer bedst til din tilstand i dag.

Markér venligst, under hver overskrift, DEN kasse, der bedst beskriver, hvordan du har det I DAG.

1) BEVÆGE MIG (gå)

- ◯ Jeg har INGEN problemer med at gå
- ◯ Jeg har NOGLE problemer med at gå
- ◯ Jeg har MANGE problemer med at gå

2) KLARE MIG SELV

- ◯ Jeg har INGEN problemer med at vaske mig eller tage tøj på
- ◯ Jeg har NOGLE problemer med at vaske mig eller tage tøj på
- ◯ Jeg har MANGE problemer med at vaske mig eller tage tøj på

3) GØRE DE TING, JEG PLEJER AT GØRE (for eksempel gå i skole, fritidsaktiviteter, sport, leg, lave ting med familien eller vennerne)

- ◯ Jeg har INGEN problemer med at gøre de ting, jeg plejer at gøre
- ◯ Jeg har NOGLE problemer med at gøre de ting, jeg plejer at gøre
- ◯ Jeg har MANGE problemer med at gøre de ting, jeg plejer at gøre

4) HAVE SMERTER ELLER UBEHAG (en væmmelig følelse i kroppen)

- ◯ Jeg har INGEN smerter eller INTET ubehag
- ◯ Jeg har NOGLE smerter eller NOGET ubehag
- ◯ Jeg har MANGE smerter eller MEGET ubehag

5) VÆRE BEKYMRET, TRIST ELLER KED AF DET

- ◯ Jeg er IKKE bekymret, trist eller ked af det
- ◯ Jeg er LIDT bekymret, trist eller ked af det
- ◯ Jeg er MEGET bekymret, trist eller ked af det

6) Vi vil gerne vide, hvor godt eller dårligt du har det I DAG. Denne linje går fra 0 til 100. 100 betyder så godt som du kan forestille dig, at man kan have det. 0 betyder så dårligt som du kan forestille dig, at man kan have det. Flyt markøren til det sted på linjen, der viser, hvor godt eller dårligt du har det I DAG.

Så dårligt som du kan forestille dig, at man kan have det — Så godt som du kan forestille dig, at man kan have det

(Place a mark on the scale above)

Spørgeskema om smerter i forbindelse med håndledsbrud

Kære [baseline_arm_1][fornavn].

I dette spørgeskema stiller vi dig spørgsmål om dine smerter.

Vær venlig at svare ved at markere det svar, der passer bedst til din tilstand i den forløbne uge.

Dette redskab kan hjælpe os med at forstå dit barns smerte. Hvert ansigt repræsenterer en person, der slet ikke har ondt, har lidt eller meget ondt.

Forklar dette til dit barn:
Ansigt 0 gør ikke ondt. Ansigt 2 gør lidt ondt. Ansigt 4 gør lidt mere ondt. Ansigt 6 gør endnu mere ondt. Ansigt 8 gør meget ondt. Ansigt 10 gør så ondt som du overhovedet kan forestille dig, selvom du ikke behøver at græde for at have denne værste smerte.

Hvilket ansigt ville du vælge for bedst at hjælpe lægerne med at forstå din smerte?

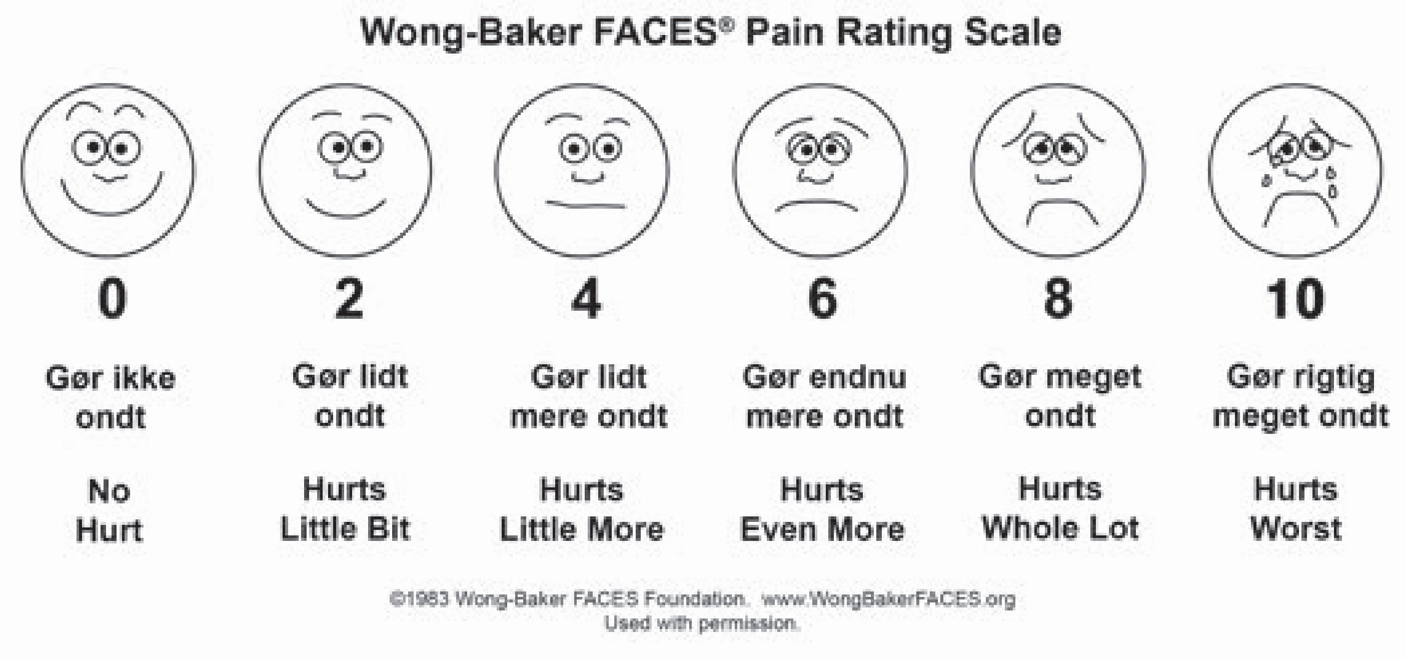

---

1) Markér venligst tallet, der svarer til det ansigt barnet peger på.

◯ 0 ◯ 2 ◯ 4 ◯ 6
◯ 8 ◯ 10

Kontrol inkl. komplikationer

Dato for besøg: ______________________

Har der været komplikationer/gener siden sidst?
- ◯ Nej
- ◯ Ja

Sæt kryds i én eller flere
- ☐ Neuropraxi
- ☐ Kosmetisk generende skævhed
- ☐ Begrænset brug af arm eller hånd (uddybes i spørgeskema)
- ☐ Smerter
- ☐ Andet

Hvilke(n)? ______________________

Sæt kryds i én eller flere
- ☐ Dyb infektion
- ☐ Neuropraxi
- ☐ Ubehagelig arvæv
- ☐ Kosmetisk generende skævhed
- ☐ Begrænset brug af arm eller hånd (uddybes i spørgeskema)
- ☐ Smerter
- ☐ Andet

Hvilke(n)? ______________________

Her kan uploades billede af sår, infektion e.lign.
hvis det er relevant for dette besøg.

Her kan uploades billede af sår, infektion e.lign.
hvis det er relevant for dette besøg.

Her kan uploades billede af sår, infektion e.lign.
hvis det er relevant for dette besøg.

Er der lavet eller planlagt osteotomi?
- ◯ Nej
- ◯ Ja

Hvilken type osteotomi? ______________________

Dato for udført/planlagt osteotomi: ______________________

Ekstra besøg generelt

1) Dato for besøg

____

2) Årsag til ekstra besøg:

____
(Beskriv kortfattet årsag til ekstra besøg,
f.eks. manglende heling, infektion, sårheling etc.
)

3) Her kan uploades billede af sår, infektion e.lign. hvis det er relevant for dette besøg.

4) Her kan uploades billede af sår, infektion e.lign. hvis det er relevant for dette besøg.

5) Her kan uploades billede af sår, infektion e.lign. hvis det er relevant for dette besøg.

Afsluttende data

Study ID

____

Oplysninger om afslutning af undersøgelsen

Har patienten gennemført studiet?

◯ Nej
◯ Ja

Dato for gennemførelse af studiet:

____
(DD-MM-YYYY)

Hvad er årsagen til at patienten ikke gennemførte?

☐ Barnet er flyttet ud af Danmark
☐ Helbredsmæssige årsager, der forhindrer deltagelse i forsøget
☐ Udeblivelse fra kontroller på trods af remindere
☐ Ønsker ikke længere at deltage (samtykke trukket tilbage)
☐ Andet
(Kryds af i én eller flere svar)

Hvis andet, hvilke(n)?

____

Dato patientens deltagelse er stoppet

____
(DD-MM-YYYY)

Generelle kommentarer

Kommentarer

____
