# Supplementary material for: Is casting of displaced paediatric distal forearm fractures non-inferior to reduction under general anaesthesia? Study protocol for a pragmatic, randomized, controlled non-inferiority multicentre trial (the casting trial)
Source: Trials. 2024 Jun 27;25:420. doi: 10.1186/s13063-024-08253-z (PMC11212181; doi:10.1186/s13063-024-08253-z)
Supplement: Supplementary file 3 — Additional file 3. Original and English translations of funding documentation. [file 13063_2024_8253_MOESM3_ESM.pdf]

Til rette vedkommende.

Dato: 13/7-2021

Journalnummer:
Initialer: TNSE

Afdeling
Ortopædkirurgisk Afdeling
SUH Køge
Lykkebækvej 1
4600 Køge
Tlf.: 47 32 33 01
koe-ortkir@regionsjaelland.dk

Direkte tlf.: 47 32 33 01
E-mail: tnse@regionsjaelland.dk

www.regionsjaelland.dk

Underskudsgaranti vedr. ph.d. forløb for:

Reservelæge Katrine Abildgaard Nielsen

Vedrørende ph.d.-projektet "Can completely displaced or overriding distal forearm fractures in children be treated without surgical reduction?" bekræftes det, at Ortopædkirurgisk Afdeling, Sjællands Universitetshospital Køge, stiller underskudsgaranti for dækning af løn til ph.d.-studerende Katrine Abildgaard Nielsen i op til 3 år fra d. 1/9-2022, såfremt der ikke kan opnås andre finansieringsmidler.

Med venlig hilsen

Sjællands Universitetshospital
Lykkebækvej 1, 4600 Køge
Ledende overlæge Thomas Juul Sørensen
Speciallæge i ortopædkirurgi
Tlf. 4732 3301
Email: TNSE@regionsjaelland.dk

Thomas Juul-Sørensen
Ledende overlæge
Ortopædkirurgisk Afdeling
Sjællands Universitetshospital Køge

July 13th 2021

To whom it may concern.

Salary covering guarantee for PhD programme for:

Resident Katrine Abildgaard Nielsen

Regarding the PhD project "Can completely displaced or overriding distal forearm fractures in children be treated without surgical reduction?" it is confirmed that the Department of Orthopaedic Surgery, Zealand University Hospital Køge, provides a deficit guarantee to cover the salary of PhD student Katrine Abildgaard Nielsen for up to 3 years from 1 September 2022, if no other funding can be obtained.

With kind regards

Thomas Juul-Sørensen

Senior Consultant

Department of Orthopaedic Surgery

Zealand University Hospital Køge
