# Supplementary material for: Is casting of displaced paediatric distal forearm fractures non-inferior to reduction under general anaesthesia? Study protocol for a pragmatic, randomized, controlled non-inferiority multicentre trial (the casting trial)
Source: Trials. 2024 Jun 27;25:420. doi: 10.1186/s13063-024-08253-z (PMC11212181; doi:10.1186/s13063-024-08253-z)
Supplement: Supplementary file 5 — Additional file 5. Original and English translation of ethical approval. [file 13063_2024_8253_MOESM5_ESM.pdf]

REGION SJÆLLAND
FORSKNINGSSTØTTE CENTER
- vi er til for dig

Katrine Rønn Abildgaard
Sjællands Universitetshospital
Ortopædkirurgisk Afdeling
Lykkebækvej 1
4600 Køge

Dato: 12. september 2023

Sags ID: EMN-2023-00723
Dokument ID: 11116852

Sekretariatet for Videnskabsetisk Komite
Lykkebækvej 1

4600 Køge
www.regionsjaelland.dk

E-mail: rvk-sjaelland@regionsjaelland.dk

Berigtiget endelig godkendelse af forsøg SJ-1026, "Is casting of displaced paediatric distal forearm fractures non-inferior to reduction under general anaesthesia? The CASTING trial. "

Afgørelsen er truffet efter lovbekendtgørelse nr. 1338 af 1. september 2020 om videnskabsetisk behandling af sundhedsvidenskabelige forskningsprojekter og sundhedsdatavidenskabelige forskningsprojekter (komitéloven).

Den Videnskabsetiske Komité for Region Sjælland bekræfter modtagelsen af mail af 15. maj 2023, som svar på komiteens afgørelse af 11. maj 2023, hvori der opstilledes betingelser for godkendelsen af projektet.

Betingelserne for godkendelsen anses for opfyldt. Projektet er dermed endeligt godkendt.

Godkendelsen gælder til den 1. april 2025 og omfatter følgende dokumenter:

- Forsøgsprotokol, version 3, af 12. juni 2023 og de til protokollen hørende spørgeskemaer,
- Deltagerinformation, version 2, af 6. juni 2023
- Informeret samtykkeerklæring, version 1, af 17. januar 2023

Godkendelsen gælder for de anmeldte forsøgssteder og den anmeldte forsøgsansvarlige i Danmark.

Det bemærkes, at komiteen ikke er ressortmyndighed for regelsættet om databeskyttelse. Komiteen forudsætter at projektet gennemføres og informationen til deltagerne gives i overensstemmelse databeskyttelsesforordningen og databeskyttelsesloven.

Vær opmærksom på, at den dataansvarlige efter databeskyttelsesforordningen har pligt til at underrette de registrerede

Region Sjælland anvender de personoplysninger, du giver os i forbindelse med din henvendelse. Du kan læse mere om formålet med anvendelsen samt dine rettigheder på vores hjemmeside nederst på regionens hjemmeside.

om, at der sker behandling af oplysninger. Databeskyttelsesforordningen indeholder en række krav til bl.a. indhold og form af underretningen. Læs mere om oplysningspligten (art. 13 og 14) og om de registreredes øvrige rettigheder i Datatilsynets vejledning om de registreredes rettigheder (se www.datatilsynet.dk).

Iværksættelse af projektet i strid med godkendelsen kan straffes med bøde eller fængsel, jf. komitélovens § 41.

Ændringer

Foretages der væsentlige ændringer i protokolmaterialet under gennemførelsen af projektet, skal disse anmeldes til komiteen i form af tillægsprotokoller. Ændringerne må først iværksættes efter godkendelse fra Komiteen, jf. komitélovens § 27, stk. 1.

Anmeldelse af tillægsprotokoller skal ske elektronisk på www.drvk.dk, ved brug af det allerede tildelte anmeldelsesnummer og adgangskode.

Væsentlige ændringer er bl.a. ændringer, der kan få betydning for forsøgspersonernes sikkerhed, fortolkning af den videnskabelige dokumentation, som projektet bygger på samt gennemførelsen eller ledelsen af projektet. Det kan fx være ændringer i in- og eksklusionskriterier, forsøgsdesign, antal forsøgspersoner, forsøgsprocedurer, behandlingsvarighed, effektparametre, ændringer om de forsøgsansvarlige eller forsøgssteder samt indholdsmæssige ændringer i det skriftlige informationsmateriale til forsøgspersonerne.

Hvor nye oplysninger betyder, at forskeren overvejer at ændre proceduren eller stoppe forsøget, skal komiteen orienteres om det.

Bivirkninger og hændelser

Løbende indberetning

Komiteen skal omgående underrettes, hvis der under projektet optræder formodet alvorlige, uventede bivirkninger eller alvorlige hændelser, jf. komitélovens § 30, stk. 1. Indberetningen skal ledsages af kommentarer om eventuelle konsekvenser for forsøget. Det er kun bivirkninger og hændelser forekommet i Danmark, der skal indberettes. Underretning skal ske senest 7 dage efter, at sponsor eller den forsøgsansvarlige har fået kendskab til tilfældet.

Ved indberetning kan anvendes et skema, der findes på www.nvk.dk. Skemaet med bilag indsendes elektronisk.

Årlig indberetning

Én gang årligt i hele forsøgsperioden skal komiteen have tilsendt en liste over alle formodet alvorlige (ventede og uventede) bivirkninger og alvorlige hændelser, som er indtruffet i forsøgsperioden sammen med en rapport om forsøgspersonernes sikkerhed, jf. komitélovens § 30, stk. 2.

Materialet skal være på dansk eller engelsk.

Ved indberetning skal anvendes et skema, der findes på www.nvk.dk. Skemaet med bilag indsendes elektronisk.

Afslutning
Den forsøgsansvarlige skal senest 90 dage efter afslutningen af projektet underrette komiteen herom, jf. komitélovens § 31, stk. 1. Projektet regnes som afsluttet, når sidste forsøgsperson er afsluttet.

Ved indberetning skal anvendes et skema, der findes på www.nvk.dk. Skemaet indsendes elektronisk.

Afbrydes projektet tidligere end planlagt, skal en begrundelse herfor sendes til komiteen senest 15 dage efter, at beslutningen er truffet, jf. komitélovens § 31, stk. 2.

Hvis projektet ikke påbegyndes, skal dette samt årsagen hertil meddeles komiteen.

Komiteen beder om kopi af den afsluttende forskningsrapport eller publikation, jf. Komitélovens § 28, stk. 2. Vi skal i den forbindelse gøre opmærksom på, at der er pligt til at offentliggøre både negative, positive og inkonklusive forsøgsresultater, jf. komitélovens § 20, stk. 1, nr. 8.

Tilsyn
Komiteen fører tilsyn med, at projektet udføres i overensstemmelse med godkendelsen, jf. komitélovens §§ 28 og 29.

Afgørelsen er berigtiget således, at :

Titlen på projektet blev ændret fra ”The CANDY trial. CAN Displaced distal forearm fractures in Young children be treated with a splint and a little patience? A randomized, controlled, non-inferiority trial. ”

til

”Is casting of displaced paediatric distal forearm fractures non-inferior to re-duction under general anaesthesia? The CASTING trial. “

Berigtiget den 11. oktober 2023
Sekretær, Trine Ziebell

Venlig hilsen

Trine Ziebell
Sekretær i Sekretariatet for den Videnskabsetiske Komité for Region Sjælland

Region Sjælland anvender de personoplysninger, du giver os i forbindelse med din henvendelse. Du kan læse mere om formålet med anvendelsen samt dine rettigheder på vores hjemmeside nederst på regionens hjemmeside.

Corrected final approval of trial SJ-1026, "Is casting of displaced paediatric distal forearm fractures non-inferior to reduction under general anaesthesia? The CASTING trial. "

The decision has been made in accordance with Consolidated Act no. 1338 of 1 September 2020 on the scientific ethical treatment of health science research projects and health data science research projects (the Committee Act).

The Research Ethics Committee for Region Zealand confirms receipt of the email of 15 May 2023, in response to the committee's decision of 11 May 2023, in which conditions were set for the approval of the project.

The conditions for approval are considered to have been met. The project is thus finally approved.

The approval is valid until 1 April 2025 and includes the following documents:

- Study protocol, version 3, of 12 June 2023 and the questionnaires associated with the protocol,
- Participant information, version 2, dated 6 June 2023
- Informed consent form, version 1, dated 17 January 2023

The authorisation applies to the notified trial sites and the notified investigator in Denmark.

It should be noted that the committee is not the competent authority for data protection regulations. The committee assumes that the project is carried out and the information to participants is provided in accordance with the General Data Protection Regulation and the Danish Data Protection Act.

Please note that according to the GDPR, the data controller is obliged to inform data subjects that data is being processed. The General Data Protection Regulation contains a number of requirements for, among other things, the content and form of the notification. Read more about the duty to inform (art. 13 and 14) and about the data subjects' other rights in the Danish Data Protection Agency's guidance on the rights of the data subjects’ rights (see www.datatilsynet.dk).

Implementation of the project in violation of the authorisation is punishable by a fine or imprisonment, cf. Section 41 of the Committees Act.

Changes to the protocol

If significant changes are made to the protocol material during the execution of the project, these must be notified to the committee in the form of supplementary protocols. The changes may only be initiated after approval from the Committee, cf. section 27(1) of the Danish Act on Committees.

Notification of supplementary protocols must be done electronically at www.drvk.dk, using the already assigned notification number and password.

Significant changes are, among other things, changes that may affect the safety of trial subjects, interpretation of the scientific documentation, on which the project is based, and the implementation or management of the project. For example, changes in inclusion and exclusion criteria, trial design, number of subjects, trial procedures, duration of treatment, efficacy parameters, changes to the investigators or trial sites as well as changes to the content of the written information material for the trial subjects.

Where new information means that the researcher is considering changing the procedure or stopping the trial, the committee must be informed.

Adverse events and incidents

Continuous reporting

The committee must be informed immediately if suspected serious unexpected adverse reactions or serious adverse events occur during the project, unexpected adverse reactions or serious adverse events, cf. section 30(1) of the Danish Committees Act. The report must be accompanied by comments on any consequences for the trial. Only adverse reactions and events occurring in Denmark must be reported. Notification must be made no later than 7 days after the sponsor or the person responsible for the trial has become aware of the event.

A form available at www.nvk.dk can be used for reporting. The form with appendices must be submitted electronically.

Annual reporting

Once a year throughout the trial period, the committee must receive a list of all suspected serious (expected and unexpected) adverse reactions and serious adverse events that have occurred during the trial period together with a report on the safety of the trial subjects, cf. section 30(2) of the 30(2) of the Committees Act.

The material must be in Danish or English.

A form available at www.nvk.dk must be used for reporting. The form with appendices must be submitted electronically.

Completion

The trial coordinator must notify the committee no later than 90 days after the end of the project, cf. section 31(1) of the Danish Committees Act. The project is considered completed when the last trial subject has been terminated.

A form available at www.nvk.dk must be used for reporting. The form must be submitted electronically.

If the project is terminated earlier than planned, a justification must be submitted to the committee no later than 15 days after the decision has been made, cf. section 31(2) of the Danish Act on Committees.

If the project is not commenced, the committee must be notified of this and the reasons for it.

The committee requests a copy of the final research report or publication cf. section 28(2) of the Danish Act on Committees. In this connection, please note that there is an obligation to publish negative, positive and inconclusive trial results, cf. section 20(1)(8) of the Danish Act on Committees.

Oversight

The Committee oversees that the project is carried out in accordance with the authorisation, cf. sections 28 and 29 of the Danish Committees Act.

The decision has been corrected so that:

The title of the project was changed from "The CANDY trial. CAN Displaced distal forearm fractures in Young children be treated with a splint and a little patience? A randomised, controlled, non-inferiority trial. "

to

"Is casting of displaced paediatric distal forearm fractures non-inferior to reduction under general anaesthesia? The CASTING trial. "

Corrected on 11 October 2023 Secretary, Trine Ziebell

Sincerely,

Trine Ziebell

Secretary in the Secretariat of the Research Ethics Committee for Region Zealand
