# Supplementary material for: Is casting of displaced paediatric distal forearm fractures non-inferior to reduction under general anaesthesia? Study protocol for a pragmatic, randomized, controlled non-inferiority multicentre trial (the casting trial)
Source: Trials. 2024 Jun 27;25:420. doi: 10.1186/s13063-024-08253-z (PMC11212181; doi:10.1186/s13063-024-08253-z)
Supplement: Supplementary file 6 — Additional file 6. Consent form. [file 13063_2024_8253_MOESM6_ESM.pdf]

Fra: Region Sjælland [mailto:no-reply@efond.dk]
Sendt: 12. januar 2023 08:45
Til: Katrine Rønn Abildgaard <kaabn@regionsjaelland.dk>; REG FORSK <reg.forsk@regionsjaelland.dk>
Emne: Region Sjællands Sundhedsvidenskabelige Forskningsfond 2023 - A1461 Stig Brorson

Forsigtig: Ekstern mail

Kære Stig Brorson

Vedr. ansøgning nr. R37-A1461 om forskningsmidler fra Region Sjællands Sundhedsvidenskabelige Forskningsfond (RSSF) 2023:

Projekttitel: 'Does treatment of Completely displAced or overridiNg Distal forearm fractures in children merelY require a splint and a little patience? A randomized, controlled, non-inferiority trial '

Fondsbestyrelsen for RSSF har behandlet din ansøgning og har truffet afgørelse om at støtte projektet med kr. 250.000.

Bevillingen kan kun bruges til det ansøgte projekt med det beskrevne formål.

Bevillingen overføres til sygehuset ved den kommende budgetoverførsel og administreres af sygehusets økonomikontor. Du bør tage kontakt til sygehusets økonomikontor inden 14 dage og oplyse om den modtagne projektbevilling. Sygehusets økonomikontor opretter en projektkonto, hvor projektets udgifter konteres. Projektet vil indgå i afdelingens budget. Projektets udgifter skal godkendes af afdelingsledelsen før fremsendelse til kontering i økonomikontoret. Der henvises i øvrigt til regionens kasse- og regnskabsregulativ.

Ved årsskifte har afdelingsledelsen og økonomikontoret ansvar for, at overføre saldoen på projektet til næste års budget.

Ved afslutning af projektet har afdelingsledelsen og økonomikontoret ansvar for at uforbrugte midler tilbageføres til Data og udviklingsstøtte. Dette gøres i forbindelse med årsafslutningen.
Ved afslutning af projektet skal slutrapport udfyldes via efond www.efond.dk/rsj

Har du spørgsmål til bevillingen, er du velkommen til at henvende dig på e-mail: reg.forsk@regionsjaelland.dk

Region Sjælland tilbyder support til at finde og søge forskningsmidler. Du kan tage kontakt til Region Sjællands Fondskontor på fondskontor@regionsjaelland.dk.

Med venlig hilsen

Bo Borg Mikkelsen
Chefkonsulent, Data og udviklingsstøtte

January 12th 2023

Dear Stig Brorson

Regarding application no. R37-A1461 for research funding from Region Zealand Health Science Research Foundation (RSSF) 2023:

Project title: 'Does treatment of Completely displAced or overridiNg Distal forearm fractures in children merelY require a splint and a little patience? A randomised, controlled, non-inferiority trial'

The RSSF Foundation Board has reviewed your application and has decided to support the project with DKK 250,000.

The grant can only be used for the project applied for with the described purpose.

The grant will be transferred to the hospital in the upcoming budget transfer and is administered by the hospital's finance office. You should contact the hospital's finance office within 14 days and inform them of the received project grant. The hospital's finance office will create a project account where the project's expenses are posted. The project will be included in the department's budget. The project expenses must be approved by the department management before being forwarded for posting to the finance office. Please also refer to the region's cash and accounting regulations.

At the end of the year, the department management and the finance office are responsible for transferring the balance of the project to the next year's budget.

At the end of the project, department management and the finance office are responsible for ensuring that unused funds are returned to Data and Development Support. This is done in connection with the year-end closing. At the end of the project, the final report must be completed via efond www.efond.dk/rsj

If you have any questions about the grant, you are welcome to contact us by email: reg.forsk@regionsjaelland.dk

Region Zealand offers support for finding and applying for research funding. You can contact Region Zealand's Foundation Office at fondskontor@regionsjaelland.dk.

With kind regards

Bo Borg Mikkelsen Chief Consultant, Data and Development Support

Katrine Rønn Abildgaard

Fra: Region SJ <no-reply@efond.dk>
Sendt: 29. august 2022 07:08
Til: Katrine Rønn Abildgaard; REG FORSK
Emne: Vedr. ansøgning A1457 til Region SJ

Forsigtig: Ekstern mail

Kære Katrine Rønn Abildgaard

Vedr. din ansøgning nr. R24 A1457 med titlen:
Does treatment of completely displaced or overriding distal forearm fractures in children merely require a splint and a little patience?

Data og udviklingsstøtte (DU) har behandlet din ansøgning om dækning af studieafgift for 1., 2. og 3. år og annuum og har bevilget per år: 50.000 kr. til studieafgift og 20.000 kr. til annuum.
Bevillingen må kun bruges til det projekt og de formål, der er ansøgt om og bevilget støtte til.

Venligst informer din afdelingsledelse om bevillingen.

Studieafgift: Hvis du ikke allerede har gjort det, skal du snarest tage kontakt til det universitet, hvor du er indskrevet og oplyse EAN-nr. i forbindelse med E-fakturering af studieafgift:
EAN 5798002040906
Att. Bo Borg Mikkelsen, Data og udviklingsstøtte

Det er således Data og udviklingsstøtte der betaler studieafgiften via faktura fra universitetet.

Annuum: Beløbet overføres Fra Region Sjælland til sygehuset ved budgettilførsel og bliver administreret via sygehusets lokale økonomiafdeling. Du skal derfor straks tage kontakt til dit lokale økonomikontor og oplyse dem om din bevilling. Økonomikontoret vil herefter sørge for at oprette en projektkonto til dig, der hører under din afdelings budget, og udgiftsbilag vedr. dit projekt skal derfor påtegnes af din afdelingsledelse før fremsendelse til Regnskab.

Da bevillingen dermed bliver budgettilført din afdeling, er det også din afdelingsledelse, der inden årets udgang skal sørge for at bede om at få overført evt. ubrugte midler til næste års budget. Evt. restbeløb skal tilbageføres til Region Sjælland. I tilfælde af du ikke færdiggør dit studie, skal midlerne fra de ikke færdiggjorte år tilbageføres til regionen.

Når du har afsluttet dit ph.d.-forløb skal du afgive en slutrapport og regnskab via din adgang til efond, hvor du også har indsendt dine ansøgninger om studieafgift.

Hvis du har spørgsmål til ovenstående, er du meget velkommen til at kontakte DU via reg.forsk@regionsjaelland.dk

Har du behov for hjælp til at finde finansiering til forskning, kan du kontakte Fondskontoret i Region Sjælland: https://www.regionsjaelland.dk/Udvikling/Fondskontoret

Med venlig hilsen
Bo Borg Mikkelsen, Chefkonsulent, Data og udviklingsstøtte

August 29th 2022

Dear Katrine Rønn Abildgaard.

Regarding your application no. R24 A1457 with the title:

Does treatment of completely displaced or overriding distal forearm fractures in children merely require a splint and a little patience?

Data and Development Support (DU) has processed your application for coverage of tuition fees for 1st, 2nd and 3rd year and annuum and has granted per year: DKK 50,000 for tuition fees and DKK 20,000 for annuum.

The grant may only be used for the project and purposes for which funding has been applied for and granted.

Please inform your department management about the grant.

Tuition fee: If you have not already done so, you must contact the university where you are where you are enrolled and provide your EAN no. in connection with E-invoicing of tuition fees: EAN 5798002040906 Attn. Bo Borg Mikkelsen, Data and Development Support

It is Data and Development Support that pays the tuition fee via invoice from the university.

Annuum: The amount is transferred from Region Zealand to the hospital by budget transfer and is administered via the hospital's local finance department. You must therefore immediately contact your local finance office and inform them of your authorisation. The finance office will then set up a project account for you under your department's budget, and the expense voucher for your project must therefore be endorsed by your department management before submission to Accounting.

As the authorisation is then allocated to your department's budget, it is also your department management who must before the end of the year to ask for any unused funds to be transferred to next year's budget. Any remaining amount must be returned to Region Zealand. In the event that you do not complete your study programme, the funds from the unfinished years must be returned to the region.

When you have completed your PhD programme, you must submit a final report and accounts via your access to efond, where you have also submitted your tuition fee applications.

If you have any questions about the above, you are very welcome to contact data and development support via reg.forsk@regionsjaelland.dk

If you need help finding funding for research, you can contact the Foundation Office in Region Zealand: https://www.regionsjaelland.dk/Udvikling/Fondskontoret

With kind regards

Bo Borg Mikkelsen, Chief Consultant, Data and Development Support

KONG CHRISTIAN DEN TIENDES FOND
Prins Jørgens Gård 13
1218 København K.
Tlf.: 33 63 27 50

Sekretariat:
Administrationschef Lisa Rising
Sekretær Lene Christensen

93/2023
Den 26. september 2023

Læge Katrine Rønn Abildgaard
Sjællands Universitetshospital Køge
Ortopædkirurgisk Afdeling

Fondens bestyrelse har på et bestyrelsesmøde vedtaget at yde Dem støtte til det ansøgte formål med 50.000 kr.

Beløbet vil blive fremsendt fra Danske Forvaltning.

Med venlig hilsen

Jens Peter Christensen

KING CHRISTIAN THE TENTHS FUND
Prins Jørgens Gård 13
1218 København K.
Tlf: 33 63 27 50

Secretarial office:
Head of administration Lisa Rising
Secretary Lene Christensen

September 26th 2023

Doctor Katrine Rønn Abildgaard

Zealand University Hospital Køge

Department of Orthopaedic Surgery

At a board meeting, the foundation's board of directors has decided to grant you support for the purpose with DKK 50,000.

The amount will be forwarded from Danske Forvaltning.

With kind regards

Jens Peter Christensen

Jes Bruun Lauritzen
Professor, dr. med.
Speciallæge i ortopædisk kirurgi

København, den 27. september 2023

Kære Thomas Juul Sørensen.

Tak for din indstilling af Katrine Rønn Abildgaard til en donation fra Guildal Fondet.

Hun er blevet tildelt en donation fra fondet på kr. 8.000 og vil modtage denne ved DOS Kongressen 2023, fredag den 17. november 2022.

Guildal Fondet har indført samme princip som Dansk Ortopædisk Selskabs Fond vedrørende manglende fremmøde ved uddelingen, jvf. DOS Bulletin nr. 4. juni 2006, side 15. Det vil sige, at hvis ikke man har meddelt, at man ikke kan møde op grundet vagt eller andet gyldigt forfald, og ikke sender en stedfortræder, der kan modtage legatet, så går dette tilbage til Fondet.

Med venlig hilsen
f./Jes Bruun Lauritzen
professor, dr. med.
Janne Elholm, lægesekr.
tlf. 26 29 97 22

Copenhagen, September 27th 2023

Dear Thomas Juul Sørensen.

Thank you for your nomination of Katrine Rønn Abildgaard for a donation from the Guildal Foundation.

She has been awarded a donation from the foundation of DKK 8,000 and will receive this at the DOS Congress 2023, Friday 17 November 2022.

The Guildal Foundation has introduced the same principle as the Danish Orthopaedic Society Foundation regarding non-attendance at the award ceremony, cf. DOS Bulletin no. 4 June 2006, page 15. This means that if you have not notified us that you are unable to attend due to on-call duty or other valid absence, and do not send a substitute who can receive the scholarship, then this will be returned to the Foundation.

With kind regards

f./Jes Bruun Lauritzen

Professor, dr. med.

Janne Elholm, medical secretary.

tel. +45 26 29 97 22
